# Accurate and cost-effective workflow integrating trio pooled-WES for novel gene discovery in neurodevelopmental disorders

**DOI:** 10.1038/s41431-026-02075-0

**Published:** 2026-03-14

**Authors:** Lucía López-López, Laura Lapeña-Gil, Yolanda Benítez, Caridad Serrano, Ana Isabel Sánchez-Barbero, Fiona Blanco-Kelly, Fermina López-Grondona, Saoud Tahsin-Swafiri, Isabel Lorda-Sánchez, Carmen Ayuso, Pablo Mínguez, Berta Almoguera

**Affiliations:** 1 Department of Genetics & Genomics, Health Research Institute-Fundación Jiménez Díaz University Hospital, Universidad Autónoma de Madrid (IIS-FJD, UAM), Madrid, Spain; 2 Center for Biomedical Network Research on Rare Diseases (CIBERER), Instituto de Salud Carlos III, Madrid, Spain; 3 Bioinformatics Unit, Health Research Institute-Fundación Jiménez Díaz University Hospital, Universidad Autónoma de Madrid (IIS-FJD, UAM), Madrid, Spain

**Keywords:** Genetic testing, Neurological disorders

## Abstract

The broad genetic heterogeneity of neurodevelopmental disorders (NDDs) makes their molecular diagnosis particularly challenging. In this context, Whole-Exome Sequencing (WES), specifically in a trio-based design, is a powerful strategy due to its ability to detect de novo variants, which are a major contributor to NDDs. However, its clinical implementation is often limited by its associated cost. In this study, we applied a sequential diagnostic workflow to a cohort of 221 individuals with syndromic NDDs and prior negative results from targeted sequencing. The workflow integrates initial solo-WES, followed by a second-tier trio-WES using pooled parental DNA (trio pooled-WES). Overall, this workflow achieved a diagnostic yield of 20.98% and led to the identification of 13 novel candidate genes. The pooling strategy was optimized and validated, demonstrating that trio pooled-WES retains the main advantages of conventional trio-WES while substantially reducing sequencing costs. These results support its implementation as a clinically applicable approach for the genetic diagnosis of NDDs.

## Introduction

Neurodevelopmental Disorders (NDDs) encompass a broad range of conditions that affect brain development, resulting in cognitive, motor and social impairments [1]. The heterogeneity of NDDs is evident across clinical and phenotypic dimensions [2], reflecting the complexity of their underlying genetic architecture. Virtually any type of genetic variation is involved in NDDs [3, 4], and more than 1500 genes have already been unequivocally implicated in their pathogenesis (PanelApp, last accessed July 2025). Yet, Kaplanis et al. [5] estimated that more than 1000 additional genes remain undiscovered.

A key driver of progress in NDD diagnostics has been next-generation sequencing (NGS). Among the techniques that NGS encompasses, Whole-Exome Sequencing (WES) has emerged as the first-tier diagnostic tool, offering a final mean diagnostic yield of 30–50% [6, 7]. In NDDs, the effectiveness of WES is enhanced using a trio-based approach (trio-WES), which sequences both parents and the affected child. By adding information about the parents’ genotype, it directly enables the detection of de novo variants, which is the most prevalent pathogenic mechanism in these disorders [8, 9]. This strategy facilitates filtering, interpretation, and prioritization of variants, improving the discovery of novel causative genes [10]. However, the cost associated with a trio-WES is still a limitation for universal clinical implementation in routine genetic diagnosis [11].

To address this limitation, an alternative approach using pooled DNA sequencing has been proposed [12]. This method involves the sequencing of a number of DNA samples of fathers and mothers of index cases in two separate pools. The approach is solely used to ascertain the presence or absence of the variant within the parental pools, conserving the advantages of trio-WES analysis while maintaining a lower overall cost. Despite the promising potential of this technique, it remains underutilized in clinical settings.

In this study, we propose the use of a sensitive sequential workflow for the identification of rare and novel genetic causes of monogenic NDDs. Our study aims to establish the optimal conditions for the clinical implementation of the pooling approach, assessing the technical efficiency of this strategy, and demonstrating its value in identifying novel causative genes in NDDs.

## Materials and methods

### Patient selection and sample preparation

This project was approved by the Ethics Committee of Fundación Jiménez Diaz University Hospital (FJD-UH; PIC202-23_FJD) and conducted following the principles stated in the Declaration of Helsinki as well as institutional requirements. Written informed consent was obtained from all participants or their legal guardians.

Patients were selected from the NDD cohort at FJD-UH. This cohort includes over 3000 patients with an NDD, studied primarily with targeted sequencing (TS) with the Clinical Exome Solution v2 or v3 from SOPHiA Genetics (SOPHiA Genetics, Boston) that captures 4500 to 5000 genes (depending on the version) involved in human genetic conditions. A subset of these patients is also studied with array CGH (prior to TS), Fragile-X syndrome or other techniques, depending on the diagnostic suspicion. For the present study, patients referred to the Genetics Department between 2017 and 2025 with syndromic or severe forms of NDDs and non-informative results from TS were considered. Clinical classification of the cohort was performed according to the criteria previously published by our group [13], with the addition of a category for patients presenting with NDD solely accompanied by brain malformations.

Genomic DNA from patients and their parents was extracted from EDTA-collected peripheral blood samples using automated DNA extractors: BioRobot EZ1 (QIAGEN, Hilden, Germany) or MagNA Pure Compact system (Roche Applied Science, Penzberg, Germany). DNA concentrations from parental samples were measured using the Qubit dsDNA BR Assay Kit on a Qubit 2.0 fluorometer. For the pooled-WES, equimolar amounts of DNA from samples of mothers and fathers were combined into separate pools.

### Overview of the diagnostic workflow design

The workflow used in the study was sequential and starts with a solo-WES with a regularly updated virtual panel, the NDD-FJD-Panel, that includes (1) all NDD-associated genes from the Deciphering Developmental Disorders project (DDD, https://www.deciphergenomics.org/ddd/overview), regardless of the strength of the association; (2) all genes listed in the Genomics England PanelApp for intellectual disability (green, amber and red; https://panelapp.genomicsengland.co.uk/); and (3) all newly described NDD genes from PubMed (last accessed on 18/01/2024). In patients with a clear pathogenic/likely pathogenic variant or a highly compelling variant of unknown significance (VUS), according to the ACMG guidelines [14], familial segregation was performed by Sanger sequencing, when possible. All patients with negative results from the solo-WES or with more than one VUS to segregate, and with the availability of parental DNA samples, were subjected to trio pooled-WES.

### Whole exome sequencing

WES was carried out using two different libraries: the Agilent SureSelect v6 (Agilent Technologies, Santa Clara, CA, USA) or the Twist Human Core Exome (Twist Bioscience). Pools containing the parental DNA samples were sequenced with the same library as the probands’. Paired-end sequencing (2 × 150 bp) was conducted on either NovaSeq 6000 or X systems (Illumina, San Diego, CA, USA).

For standard WES, target coverage was 100X or 200X, for Agilent SureSelect v6 and Twist Human Core Exome, respectively. For pooled-WES, target coverage was calculated to achieve a minimum of 20 reads on average for a heterozygous variant per patient following the formula Depth = (n samples in the pool * 2 alleles) * 20 reads.

### NGS data analysis

FASTQ files from solo-WES were analyzed with the FJD-Pipeline (https://github.com/TBLabFJD/PARROT-FJD) that uses Burrows-Wheeler Aligner (BWA) for sequence alignment. For small variants (single-nucleotide variants and indels, hereafter referred to as SNVs) calling, we employed an integrated approach combining HaplotypeCaller and DRAGEN from Genome Analysis Toolkit (GATK) [15] and DeepVariant [16]. SNVs annotation was performed using Variant Effect Predictor (VEP) [17] and custom sources implemented in the FJD-Pipeline. For copy number variations (CNVs), we used ExomeDepth, Convading, and Panelcn.mops for the variant calling and AnnotSV for the annotation. Other types of variants (mitochondrial or short tandem repeats) were not systematically explored.

Pooled-WES were also aligned using the FJD-Pipeline, and Mutect2 [18] was applied for the calling of SNVs. Data from the VCF files from probands and parents generated with the pipeline were combined for the trio analysis. Only SNVs with a variant allele fraction (VAF) ≥ 0.3 and a depth (DP) ≥ 20 were considered for further analyses.

### Optimization and validation of the pooling strategy

For the optimization of the pooling strategy, we confirmed all apparently de novo SNVs by Sanger sequencing and established the VAF threshold for these variants and the limit of detection of the pooled-WES.

For validation, we used an independent cohort of 16 existing parental WES to perform pooled-WES and compare the results of both approaches. Ultrarare SNVs [19], which are SNVs present in gnomADv4.1.0 with a minor allele frequency (MAF) < 0.0001, in the standard WES were assessed in the parental pools. As these variants are very rare in the general population, it is expected that each parental pool would contain the variant in only one individual (singleton). Based on this assumption, the mean allelic balance was calculated. The expected allelic balance of the pool was calculated using the following formula: *N* = 1/(n samples in the pool *2 alleles) [12]. Sensitivity (Se = [True positive/(True positive + False negative)] *100) [20] of the variant detection by pooled-WES compared with standard WES on the same samples was also assessed. Due to the nature of our pooling approach, the calculation of Specificity (S = [True negative/(True negative + False positive)] *100) was not possible, as false positives would correspond to (1) technical artifacts inherent to sequencing, which are equally likely to occur in individual WES as in pooled WES, or (2) false positives that could arise from variants present in other individuals within the pool, whose probability is extremely low for rare variants and could be safely considered zero. Therefore, specificity could be considered close to 100%.

### Single-nucleotide variant analysis

SNVs filtering was performed with PriorR (https://github.com/TBLabFJD/PriorR) [21]. For autosomal dominant (AD) and X-linked (XL) genes, SNVs were considered if the maximum allele count in gnomADv4.1.0 was <5, while for autosomal recessive (AR) genes, only SNVs with a MAF < 0.1% were included. ClinVar classifications were also used for filtering out benign/likely benign SNVs and to keep all pathogenic and likely pathogenic SNVs regardless of their MAF.

SNVs prioritization was done using the in silico predictors Combined Annotation Dependent Depletion (CADD) > 20, REVEL > 0.5, AlphaMissense > 0.6 and SIFT < 0.01 to assess exonic SNVs, or SpliceAI > 0.6 to assess the pathogenicity of synonymous and splicing SNVs. When available, the genotype of the parents from the pooled-WES was also used for prioritization.

For the identification of SNVs in novel genes potentially involved in NDDs, the parents’ genotype and additional evidence such as the allele count in gnomADv4.1.0, the Domino score [22], gene-level constraint metrics (pLI, pNull and LOEUF for loss-of-function (LoF) SNVs [23, 24] and Z scores for missense [25]), gene expression data (GTEx), and gene ontology were considered for the interpretation. No strict cutoffs of these metrics were applied; however, genes showing values closer to the established thresholds (pLI > 0.9, pNull < 0.1, LOEUF < 0.6, or missense Z-score > 3.09) were given higher interpretative weight.

All SNVs meeting the following criteria were considered: (1) apparently de novo SNVs in known AD or XL genes, as well as in candidate genes with a Domino score ≥0.6 (suggestive of AD inheritance); (2) hemizygous SNVs in XL genes; and (3) homozygous or compound heterozygous SNVs in known AR genes or in candidate genes with a Domino score <0.6 (suggestive of AR inheritance).

### Copy number variation analysis

CNV analysis was performed on solo-WES data. We considered exclusively those CNVs overlapping at least one gene from our NDD-FJD-Panel. Filtering criteria focused on quality and consistency: CNVs detected in multiple individuals from the same sequencing run were removed, as were those identified by a single caller. CNVs with LOEUF < 0.6 (indicative of low functional impact) were also excluded. For deletions, we applied an additional filter based on the haploinsufficiency score (HI < 40) and discarded events fully contained within deletions reported in the control population. For duplications, we filtered based on triplosensitivity scores (TS < 40) and excluded those contained in population benign duplications. After filtering, we prioritized CNVs supported by >1 caller, affecting coding regions, and involving genes with phenotypic relevance consistent with the patient’s clinical presentation.

## Results

### Workflow results

**Fig. 1:**
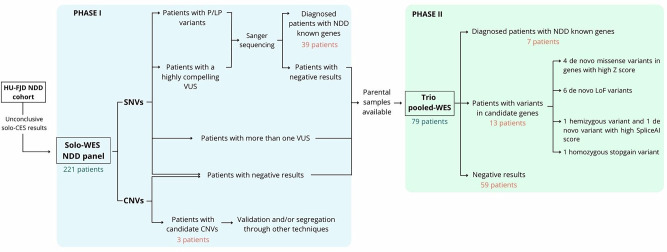
Schematic overview of the diagnostic workflow followed in this study. Patients from the HU-FJD NDD cohort with non-informative results from CES were considered. Those presenting with syndromic or severe forms of NDDs and clinical suspicion of a monogenic disorder entered the workflow. The first phase involved solo-WES with the NDD-FJD-Panel. Variants classified as pathogenic (P), likely pathogenic (LP), or VUS with potential relevance were validated and segregated by Sanger sequencing. Unsolved cases or those with multiple VUS proceeded to trio pooled-WES, provided parental samples were available. This second phase enabled further diagnoses, identification of candidate genes, or classification as non-solved. The number of patients entering each phase is indicated in blue, while final outcomes are annotated in orange.

A total of 221 patients from the FJD-UH cohort, presenting with syndromic or severe NDD and not solved with TS, were included in the workflow (Fig. 1).

Of the 221 patients included, only 43 (19.46%) presented with isolated moderate or severe NDD. The majority of patients were classified as dysmorphic or syndromic forms (n = 153, 69.23%), defined by the presence of >5 facial dysmorphisms and/or ≥2 comorbidities such as hearing loss, epilepsy, cardiac, renal, or ocular malformations [13]. In addition, 1 patient had NDD with macrocephaly, and 2 had NDD with microcephaly. A total of 20 patients presented with epileptic encephalopathy or NDD associated with epilepsy, and 16 showed NDD and brain malformations.

In a first step, solo-WES was performed on all 221 patients and analyzed using the NDD-FJD-Panel that comprises 3053 genes from the DDD, PanelApp and PubMed (Supplemental material). The analysis of the SNVs led to a genetic diagnosis in 39 cases (17.65%) (Supplementary Table S1). CNV analysis led to the identification of five CNVs. One had been previously detected by array CGH and was classified as pathogenic with incomplete penetrance, and therefore not considered causative of the patient’s phenotype. Another CNV affects a recessive gene but lacks a second pathogenic allele. Consequently, only three CNVs remain as candidate variants which need to be validated and/or segregated through other techniques (Supplementary Table S2).

Of the remaining 182 patients not characterized by the solo-WES, 79 had parental DNA samples available and were subjected to trio pooled-WES. For the pooled-WES, an initial pool including five mothers and five fathers was generated as a proof of concept. After confirming that this approach allowed accurate SNVs detection, additional pools were tested with eight and ten individuals to evaluate performance and cost-efficiency. The configuration with eight individuals per pool provided the best balance between data quality and sequencing cost; therefore, this format was selected for the remaining seven parental pools, with mothers and fathers always combined in separate pools. In all cases, the sequencing depth was adjusted according to the number of individuals per pool to ensure an average target coverage of at least 20 reads per heterozygous variant per sample.

### Optimization and validation of the trio pooled-WES strategy

The trio pooled-WES strategy was optimized for de novo SNVs detection with the determination of the VAF threshold to consider a variant absent in the pooled-WES. To determine the sensitivity of the approach, the results from the pooled-WES were compared with data from standard WES of the same samples.

#### Determination of the VAF threshold in the pooled-WES for the identification of de novo SNVs

The application of the filtering algorithm in the 79 trios that were subjected to pooled-WES resulted in 27 de novo SNVs that were absent in the pools in the analysis performed with Mutect2. Visualization of the SNVs in the parental pools using IGV revealed that 7 were present with a VAF ranging from 1% to 5%, 5 were present in the pools with a VAF below 1%, and 15 were absent from the parental pools. Sanger sequencing confirmed 19 SNVs as de novo (70.4%) and 8 as inherited (29.6%), consistent with the presence of the 7 SNVs above 1% VAF and one variant at 0.56% VAF (1/180 reads). Notably, none of the 19 confirmed de novo SNVs displayed a VAF higher than 0.58% (1/172 reads) in the DNA pools. Consequently, a threshold of 1% VAF in IGV was established for the identification of de novo SNVs in the pooled-WES.

#### Comparison of SNVs detection of pooled-WES versus standard WES: determination of sensitivity and allelic balance

We conducted a comparison of ultra-rare SNVs from 16 parents (8 mothers and 8 fathers) who had existing WES data and who were subjected to pooled-WES to calculate the sensitivity and the allelic balance of singleton variants in the pools.

1127 ultra-rare SNVs were identified in the 16 WES. Of those, 1091 SNVs were present in the pools with a VAF > 1% (96.81%), 18 were present in 0.55–1% of the reads in the pool, 2 in a range of 0.2–0.5% and 16 had no reads with the SNVs in the pools (1.42%). Altogether, with a limit of 1% VAF, the sensitivity of the pooling strategy was calculated to be 96.81%.

The pools in which these samples were contained included 10 individuals each; therefore, the expected allelic balance for a heterozygous variant was 5%. Across the 1127 SNVs assessed, the VAF values did not follow a normal distribution, and the median VAF was 4.74%. A Wilcoxon signed-rank test was applied and indicated no significant deviation from the expected value of 5% (*p* = 0.4340).

### Molecular findings of the trio pooled-WES

The analysis of the 79 trio pooled-WES led to the classification of 7 SNVs in known AD NDD genes as pathogenic or likely pathogenic after being confirmed as de novo variants (8.86% of the patients studied by trio pooled-WES) (Supplementary Table S3) and identified 13 patients with candidate SNVs in genes not yet associated with NDDs (16.46%): 11 with a confirmed de novo heterozygous SNVs in a likely dominant gene (Domino score ≥ 0.6) or heterozygous in an XL gene; one with a hemizygous maternal SNVs in a XL gene; and one with a homozygous LoF SNVs in a gene with a Domino score of 0.22 and LOEUF = 0.64. All these SNVs have a CADD ≥ 23 and a REVEL ≥ 0.6 or a SpliceAI≥0.6, and a MAF < 0.00001 in the population databases (Table 1). All candidate genes were uploaded to GeneMatcher [26] and are currently under validation. Therefore, the specific SNVs remain confidential and are not displayed.

**Table 1:** Candidate genes identified by trio pooled-WES in this study.

Gene	Zigosity	Inheritance	Variant Type	gnomAD MAF	Domino Score	In silico predictors						Gene intolerance scores		Rationale for inclusion as a candidate gene
						REVEL	CADD	AlphaMissense	SIFT	SpliceAI	PhyloP	pLi	Missense Z score	
***APOO***	Heterozygous	De novo	Stopgain	0.000000912	–	–	38	–	–	–	3.95	–	–	De novo LoF variant, observed in a single individual in gnomAD, within an X-linked gene previously suggested as a candidate for NDD [42].
***CCR7***	Heterozygous	De novo	Missense	–	0.7	0.73	26.8	0.986	0	–	7.84	–	2.69	De novo missense variant in a likely dominant gene; unanimously predicted deleterious, absent from gnomAD, and affecting a highly conserved residue
***CDC42BPA***	Heterozygous	De novo	Frameshift	–	0.6	–	–	–	–	–	9.325	1	–	De novo LoF variant in a likely dominant, haploinsufficient gene, absent from gnomAD.
***MAPK7***	Heterozygous	De novo	Missense	0.00000137	0.9	0.82	27	0.993	0	–	5.796	–	2.09	De novo missense variant in a likely dominant gene; unanimously predicted deleterious, observed in a single individual in gnomAD, and affecting a highly conserved residue
***MED1***	Heterozygous	De novo	Frameshift	–	0.9	–	–	–	–	–	9.633	1	–	De novo LoF variant in a likely dominant, haploinsufficient gene, absent from gnomAD.
***PITPNM2***	Heterozygous	De novo	Missense	–	0.7	0.6	25.3	0.777	0	–	7.803	–	4.33	De novo missense variant in a likely dominant gene, intolerant to missense variation. The variant is unanimously predicted to be deleterious by in silico tools, absent from gnomAD, and affects a highly conserved residue.
***PTPRT***	Heterozygous	De novo	Missense	–	0.9	0.85	24.9	0.989	0.01	–	9.809	–	3.22	De novo missense variant in a likely dominant gene, intolerant to missense variation. The variant is unanimously predicted to be deleterious by in silico tools, absent from gnomAD, and affects a highly conserved residue.
***RYR3***	Heterozygous	De novo	Splicing	–	0.9	–	34	–	–	0.96	9.993	1	–	De novo splice-site variant, absent from gnomAD and predicted to alter splicing at a highly conserved residue. The variant is located in a likely dominant, haploinsufficient gene previously proposed as a candidate for NDD [43]
***TRADD***	Heterozygous	De novo	Stopgain	–	0.8	–	35			–	0.773	0.01	–	De novo LoF variant in a likely dominant gene. The variant is absent from gnomAD.
***ZBTB17***	Heterozygous	De novo	Frameshift	–	0.9	–	–	–	–	–	5.478	1	–	De novo LoF variant in a likely dominant, haploinsufficient gene, absent from gnomAD.
***ZNF608***	Heterozygous	De novo	Stopgain	–	0.9	–	39	–	–	–	2.409	1	–	De novo LoF variant in a likely dominant, haploinsufficient gene, absent from gnomAD.
***ZNF75D***	Hemizygous	Maternal	Splicing	–	–	–	23.2	–	–	0.6	0.687	–	–	Hemizygous splicing variant in an X-linked gene, inherited from the mother. In silico predictors indicate an alteration of splicing, and the variant is absent from gnomAD.
***ANGPTL2***	Homozygous	Biallelic	Stopgain	0.00000205	–	–	44	–	–	–	2.776	0.01	–	Homozygous LoF variant in a likely recessive gene; low pLI (haploinsufficiency metric, not indicative of recessive genes). No LoF variants reported in homozygosity in gnomAD.

Overall, the application of our sequential workflow of solo-WES followed by trio pooled-WES led to 46 patients of the 221 diagnosed with a known NDD gene (20.81%), three patients with a compelling CNV (1.36%) and 13 patients (5.88%) with compelling SNVs in a novel gene.

## Discussion

This study presents a sensitive sequential workflow for identifying rare and novel genetic causes of monogenic NDDs. This approach not only enhances the diagnostic yield of NDDs but also facilitates the discovery of new genes associated with these disorders. By using WES followed by trio pooled-WES analysis, we maximize the detection of potential pathogenic variants, especially de novo, improving the genetic characterization of NDDs while maintaining a reduced cost.

WES, particularly in a trio-based design, has been extensively validated as a highly effective approach for the molecular diagnosis of NDDs [27, 28]. However, the application of trio-WES in routine clinical practice is still limited by its associated costs [11, 29]. One suggested solution to this limitation is the use of pooled-samples strategies, which enable genotyping of parental samples while significantly reducing sequencing expenses [12]. Despite their potential, to the best of our knowledge, these approaches remain underutilized in clinical practice and are rarely reported in the literature.

In our study, we implemented a sequential diagnostic workflow combining solo-WES as the first-tier approach and a trio pooled-WES strategy as the second tier. This stepwise design allowed us to optimize resource utilization, minimize unnecessary testing, and achieve a cost-efficient workflow without compromising diagnostic yield.

For the trio pooled-WES, 8–10 parental samples were combined and sequenced, targeting a theoretical minimum average coverage of 20X per heterozygous variant per individual. This design resulted in a reduction in sequencing costs compared to standard trio-WES, since in our strategy, only the proband is sequenced individually, while parental samples are grouped into independent pools. Even though the increased sequencing depth raises the cost of pooled-WES by ~28% compared to standard WES (based on internal costs), the inclusion of at least eight individuals per pool reduces the sequencing cost per parent to 1/8. Therefore, the estimated overall cost reduction is approximately 56% per trio compared to standard trio-WES. The total cost is comparable to that of a solo-WES followed by a single variant segregation analysis by Sanger sequencing, while preserving the key benefits of trio-based analysis. These include reliable identification of de novo variants, accurate phasing of compound heterozygous variants, and faster turnaround times in comparison with solo WES followed by multiple Sanger segregations, as it allows direct assessment of inheritance patterns. Importantly, once the pooling analysis pipeline is implemented, the interpretation and annotation processes become highly streamlined, as the parental genotypic information is automatically integrated into the proband’s data. Thus, the analytical workload remains comparable to that of conventional trio analysis.

To ensure analytical robustness, we optimized and validated the technical aspects of the trio pooled-WES, particularly to enhance de novo SNVs detection. The VAF detection threshold in the parental pools was set at 1% to account for the VAF found on IGV of Sanger-confirmed SNVs. This threshold reliably captured all true de novo SNVs while minimizing false positives, defined in this context as SNVs initially classified as de novo due to their absence in the parental pools, but subsequently confirmed as inherited. Furthermore, the only study that applied a parental pooling strategy comparable to ours also selected this VAF threshold based on their laboratory experience [12]. Validation, conducted through a direct comparison with standard trio-WES, revealed only a marginal reduction in sensitivity, confirming the results obtained in previous articles [12]. Of the 1127 ultra-rare SNVs identified through standard WES, only 36 (3.19%) were not detected in the pooled samples at VAF ≥ 1%. Notably, 27 of these 36 SNVs were present in six of the 16 individuals sequenced by both approaches. This could be explained by the inherent nature of the pooling technique. Minor variations during the pool preparation can lead to unequal representation of individual samples, which may result in sequencing errors or reduced sensitivity in detecting low-frequency variants.

Trio-WES has reported diagnostic yields ranging from 40% to 65% in NDD cohorts [26, 27, 29], depending on factors such as cohort NDD phenotype, presence of syndromic features, and severity. When considering only SNVs, the diagnostic yield decreases to ~30–40% [27, 28, 30]. In our study, the overall diagnostic rate of the workflow was 20.81%, which can be attributed to specific features of the study design. All patients had previously undergone TS targeting over 960 known NDD-associated genes. Consequently, individuals with variants in well-established NDD genes were excluded from our study, which may have contributed to the lower diagnostic yield observed. To provide context, in a previous study from our group [13], 245 TS analyses were performed on patients from the same NDD cohort. The diagnostic yield for SNVs in this subset, focusing on syndromic or severe NDD cases (*N* = 130), which correspond to the patients included in the present study, was 27.69% (36/130). Such variants would have been equally detectable through our workflow, indicating that the reduced yield reflects patients’ pre-selection rather than a limitation of the strategy itself.

CNVs analysis led to the identification of five CNVs, three of them considered potential candidates (1.36%). Likewise, this rate is influenced by the prior CNV screening performed in all patients through TS and, in some cases, by array CGH. In our previously published cohort [13], the diagnostic rate for CNVs in syndromic and severe forms of NDDs was 9.3%, demonstrating that a substantial proportion of pathogenic CNVs have already been identified prior to exome analysis. Further confirmation of the candidate CNVs detected by solo-WES is required to validate their pathogenicity, and segregation analysis in parents to support interpretation. Such segregation cannot be directly assessed by trio pooled-WES, since the pooling strategy is not suitable for CNV detection. This limitation arises because CNV calling algorithms used on WES mainly rely on coverage depth comparisons across individual samples to detect dosage imbalances [31, 32]. In pooled samples, the sequencing reads from multiple individuals are combined, effectively averaging the signal and obscuring individual-level variations. As a result, CNVs present in only one individual within the pool are likely to go undetected. However, CNV analysis in WES itself has inherent challenges, mainly related to the reliability of CNV detection (sensitivity and specificity) rather than the determination of inheritance. In most cases, the key issue is confirming whether a CNV is real, rather than whether it is de novo or inherited. Previous comparisons of CNV calling algorithms in WES have shown a high variability between the performance of each algorithm and a high rate of false-positive predictions, with some tools reporting up to 50% false identifications, highlighting the limited reliability of CNV detection from exome data [33]. Consequently, parental genotype information would provide limited additional value, as confirmation of the CNV by an orthogonal technique and further segregation analysis are usually required to validate CNVs.

Within the workflow, by solo-WES, 39 patients (17.65%) were diagnosed with pathogenic SNVs in genes known to be associated with NDDs. These genes were either recently associated with NDDs or had only a few reported cases in the literature, such as *MSL2* [34], *UBAP2L* [35] or *GTF3C3* [36, 37], which may explain their absence from the TS panel. Subsequent trio pooled-WES analysis identified additional diagnostic SNVs in known NDD genes in 7 cases. These patients had inconclusive results after solo-WES, either due to multiple VUS or poor phenotype–genotype correlation. The information of the inheritance provided by the parental pools enabled the diagnosis, highlighting the utility of performing trio analysis in ambiguous cases.

Furthermore, trio pooled-WES also facilitated the identification of 13 novel candidate genes (16.46% of trio pooled-WES cases). Comparable diagnostic yields involving novel gene identification have been reported in previous studies, with 12–15% following TS and trio-WES [38, 39]. These findings further support the evidence that trio analysis contributes to gene association discovery, as was reported in recent studies [40].

Although whole-genome sequencing (WGS) is becoming more widely available, WES remains the first-tier diagnostic test for NDD in clinical practice due mainly to lower cost, less data volume and simpler interpretation [41]. Therefore, the trio pooled-WES approach is relevant under current clinical constraints.

In summary, we propose a sequential analysis workflow for syndromic NDDs that combines initial solo WES with a second-tier trio pooled-WES strategy that could be implemented in routine diagnostic practice. This approach enhances the diagnostic yield and preserves all the diagnostic advantages of conventional trio-WES, including improved variant interpretation and inheritance assessment, while significantly reducing costs. Beyond improving the detection of pathogenic variants, this workflow also supports gene discovery by enabling the identification of novel candidate genes, ultimately contributing to the expanding genetic landscape of NDDs.

## Supplementary information

Supplementary Table S1.

Supplementary Table S2.

Supplementary Table S3.

NDD-FJD-Panel

## Data Availability

The data supporting the findings of this study are included in the article and Supplementary Information. Additional data are not publicly available due to privacy or ethical restrictions, but can be made available by the corresponding authors upon reasonable request.
