# Supplementary material for: Accurate and cost-effective workflow integrating trio pooled-WES for novel gene discovery in neurodevelopmental disorders
Source: Eur J Hum Genet. 2026 Mar 14;34(5):675–82. doi: 10.1038/s41431-026-02075-0 (PMC13172506; doi:10.1038/s41431-026-02075-0)
Supplement: Supplementary file 4 — NDD-FJD-Panel [file 41431_2026_2075_MOESM4_ESM.docx]

*A2ML1, AAAS, AARS, AASS, ABAT, ABCA2, ABCB11, ABCB6, ABCB7, ABCC6, ABCC8, ABCC9, ABCD1, ABCD4, ABCG5, ABHD12, ABHD16A, ABHD5, ABL1, ACACA, ACAD9, ACADM, ACADS, ACADSB, ACADVL, ACAN, ACAT1, ACBD5, ACBD6, ACE2, ACER3, ACIN1, ACO2, ACOT9, ACOX1, ACOX2, ACP5, ACSF3, ACSL4, ACTA1, ACTA2, ACTB, ACTG1, ACTL6A, ACTL6B, ACVR1, ACVR2B, ACY1, ADA, ADAM22, ADAMTS10, ADAMTS18, ADAMTS9, ADAMTSL2, ADAR, ADARB1, ADAT3, ADCY5, ADD1, ADD3, ADGRG1, ADGRG4, ADGRG6, ADGRV1, ADK, ADNP, ADPRHL2, ADRA2B, ADSL, AFF2, AFF3, AFF4, AFG3L2, AFP, AGA, AGAP1, AGK, AGL, AGMO, AGO1, AGO2, AGPAT2, AGPAT3, AGPS, AGT, AGTPBP1, AGTR2, AGXT, AHCY, AHDC1, AHI1, AIFM1, AIMP1, AIMP2, AIPL1, AIRE, AK1, AK2, AKAP17A, AKAP4, AKAP6, AKR1C2, AKR1D1, AKT1, AKT2, AKT3, ALAD, ALDH18A1, ALDH1A2, ALDH1A3, ALDH3A2, ALDH4A1, ALDH5A1, ALDH7A1, ALDOA, ALDOB, ALG1, ALG11, ALG12, ALG13, ALG14, ALG2, ALG3, ALG6, ALG8, ALG9, ALKBH8, ALMS1, ALPL, ALS2, ALX1, ALX3, ALX4, AMER1, AMOTL1, AMPD2, AMT, ANAPC1, ANGPT2, ANK2, ANK3, ANKH, ANKRD11, ANKRD17, ANKRD26, ANO1, ANO10, ANO3, ANO5, ANTXR1, AP1B1, AP1G1, AP1S1, AP1S2, AP2M1, AP2S1, AP3B1, AP3B2, AP4B1, AP4E1, AP4M1, AP4S1, AP5Z1, APC2, APTX, AQP7, AR, ARCN1, ARF1, ARF3, ARFGEF1, ARFGEF2, ARG1, ARHGAP31, ARHGAP35, ARHGAP36, ARHGAP6, ARHGEF2, ARHGEF4, ARHGEF6, ARHGEF9, ARID1A, ARID1B, ARID2, ARIH1, ARL13B, ARL14EP, ARL3, ARL6, ARMC4, ARMC9, ARNT2, ARPC4, ARSA, ARSB, ARSE, ARSF, ARV1, ARX, ASAH1, ASB12, ASCC1, ASCC3, ASCL1, ASH1L, ASL, ASMT, ASMTL, ASNS, ASPA, ASPH, ASPM, ASS1, ASTN1, ASXL1, ASXL2, ASXL3, ATAD1, ATAD3A, ATCAY, ATG4D, ATG7, ATIC, ATL1, ATM, ATN1, ATOH7, ATP11A, ATP13A2, ATP1A1, ATP1A2, ATP1A3, ATP2A2, ATP2B1, ATP2B3, ATP2C2, ATP5F1A, ATP5F1D, ATP6AP1, ATP6AP2, ATP6V0A1, ATP6V0A2, ATP6V0C, ATP6V1A, ATP6V1B1, ATP6V1B2, ATP6V1E1, ATP7A, ATP7B, ATP8A2, ATP8B1, ATP9A, ATR, ATRX, ATXN1, ATXN10, ATXN2, ATXN2L, ATXN3, ATXN3L, ATXN7, AUH, AUTS2, AVP, AVPR2, AWAT2, AXIN1, B3GALNT2, B3GALT6, B3GAT3, B3GLCT, B4GALNT1, B4GALT1, B4GALT7, B9D1, B9D2, BANF1, BAP1, BAZ2B, BBS1, BBS10, BBS12, BBS2, BBS4, BBS5, BBS7, BBS9, BCAP31, BCAS3, BCKDHA, BCKDHB, BCKDK, BCL11A, BCL11B, BCOR, BCORL1, BCS1L, BDP1, BEAN1, BFSP2, BGN, BHLHA9, BICD2, BICRA, BIN1, BLM, BLOC1S1, BLOC1S6, BMP15, BMP2, BMP4, BMPER, BMPR1B, BNC2, BOLA3, BPIFB6, BPTF, BRAF, BRAT1, BRCA1, BRCA2, BRD4, BRF1, BRIP1, BRPF1, BRSK2, BRWD3, BSCL2, BSN, BSND, BTD, BTK, BUB1, BUB1B, C12orf4, C12orf57, C12orf65, C19orf12, C1QBP, C2CD3, C2orf69, C8orf37, C9orf72, CA2, CA5A, CA8, CACNA1A, CACNA1B, CACNA1C, CACNA1D, CACNA1E, CACNA1F, CACNA1G, CACNA1H, CACNA1I, CACNA1S, CACNA2D1, CACNA2D2, CACNA2D3, CACNB4, CACNG2, CAD, CAMK2A, CAMK2B, CAMK2G, CAMK4, CAMSAP1, CAMTA1, CANT1, CAP1, CAPN10, CAPN15, CAPRIN1, CAPZA2, CARS, CARS2, CASK, CASP2, CASR, CAV1, CBL, CBS, CBX1, CC2D1A, CC2D2A, CCBE1, CCDC103, CCDC114, CCDC115, CCDC174, CCDC186, CCDC22, CCDC32, CCDC39, CCDC40, CCDC47, CCDC65, CCDC78, CCDC8, CCDC82, CCDC88A, CCDC88C, CCN2, CCNA2, CCNB3, CCND2, CCNK, CCNO, CCNQ, CCT5, CD151, CD96, CD99, CDC40, CDC42, CDC42BPB, CDC45, CDC6, CDH1, CDH11, CDH15, CDH2, CDH23, CDH3, CDK10, CDK13, CDK16, CDK19, CDK5R1, CDK5RAP2, CDK8, CDK9, CDKL5, CDKN1C, CDON, CDT1, CELF2, CENPF, CENPJ, CEP104, CEP120, CEP135, CEP152, CEP290, CEP41, CEP55, CEP57, CEP63, CEP83, CEP85L, CFAP298, CFAP300, CFAP410, CFAP418, CFAP47, CFC1, CFL2, CFP, CHAMP1, CHD1, CHD2, CHD3, CHD4, CHD5, CHD7, CHD8, CHKA, CHKB, CHL1, CHM, CHMP1A, CHMP3, CHRDL1, CHRM1, CHRNA1, CHRNA2, CHRNA3, CHRNA4, CHRNB1, CHRNB2, CHRNG, CHST14, CHST3, CHSY1, CHUK, CIB2, CIC, CISD2, CIT, CKAP2L, CLCN2, CLCN3, CLCN4, CLCN5, CLCN6, CLCN7, CLCNKA, CLCNKB, CLDN11, CLDN19, CLDN5, CLIC2, CLMP, CLN3, CLN5, CLN6, CLN8, CLP1, CLPB, CLPP, CLTC, CMC4, CMIP, CNKSR1, CNKSR2, CNNM2, CNOT1, CNOT2, CNOT3, CNOT9, CNPY3, CNTN3, CNTN4, CNTNAP1, CNTNAP2, COA3, COA5, COA8, COASY, COG1, COG3, COG4, COG5, COG6, COG7, COG8, COL10A1, COL11A1, COL11A2, COL13A1, COL18A1, COL1A1, COL1A2, COL25A1, COL27A1, COL2A1, COL4A1, COL4A2, COL4A3, COL4A3BP, COL4A4, COL4A6, COL6A1, COL6A2, COL6A3, COL9A1, COL9A2, COL9A3, COLEC10, COLEC11, COMP, COPB1, COPB2, COQ2, COQ4, COQ5, COQ8A, COQ9, COX10, COX11, COX14, COX15, COX16, COX6B1, COX7B, CP, CPA6, CPAMD8, CPD, CPE, CPLANE1, CPLX1, CPS1, CPSF3, CPXCR1, CRADD, CRB1, CRB2, CRBN, CREBBP, CRELD1, CRIM1, CRIPT, CRKL, CRLF2, CRLS1, CRPPA, CRX, CRYAA, CRYAB, CRYBA1, CRYBA4, CRYBB1, CRYBB2, CRYBB3, CRYGC, CRYGD, CSDE1, CSF1R, CSF2RA, CSNK1G1, CSNK2A1, CSNK2B, CSPP1, CSTA, CSTB, CSTF2, CTBP1, CTC1, CTCF, CTDP1, CTNNA2, CTNNB1, CTNND1, CTNND2, CTNS, CTPS2, CTR9, CTSA, CTSD, CTSF, CTSK, CTTNBP2, CTU2, CUL3, CUL4B, CUL7, CUX1, CUX2, CWC27, CWF19L1, CXorf56, CXorf58, CYB5R3, CYC1, CYFIP2, CYP1B1, CYP24A1, CYP27A1, CYP2U1, CYP7B1, D2HGDH, DAB1, DACT1, DAG1, DAGLA, DALRD3, DARS, DARS2, DAW1, DBT, DCAF17, DCC, DCDC2, DCHS1, DCHS2, DCPS, DCTN1, DCX, DDB1, DDB2, DDC, DDHD1, DDHD2, DDOST, DDR2, DDX11, DDX23, DDX3X, DDX53, DDX54, DDX58, DDX59, DDX6, DEAF1, DECR1, DEGS1, DENND5A, DEPDC5, DGAT1, DGKH, DHCR24, DHCR7, DHDDS, DHFR, DHODH, DHPS, DHRS3, DHRSX, DHTKD1, DHX16, DHX30, DHX32, DHX34, DHX37, DHX9, DIAPH1, DIAPH2, DIP2B, DIPK2A, DIS3L2, DISP1, DKC1, DLAT, DLD, DLG1, DLG2, DLG3, DLG4, DLG5, DLGAP2, DLL1, DLL3, DLL4, DLX5, DMD, DMP1, DMPK, DMXL2, DNA2, DNAAF11, DNAAF3, DNAAF4, DNAAF5, DNAH14, DNAH5, DNAH9, DNAJB13, DNAJB4, DNAJC12, DNAJC19, DNAJC3, DNM1, DNM1L, DNM2, DNMT1, DNMT3A, DNMT3B, DOCK11, DOCK3, DOCK6, DOCK7, DOCK8, DOHH, DOLK, DONSON, DPAGT1, DPF1, DPF2, DPF3, DPH1, DPH2, DPH5, DPM1, DPM2, DPM3, DPP6, DPYD, DPYS, DPYSL2, DPYSL5, DRC1, DRD2, DROSHA, DSCAM, DSE, DSG1, DSP, DSPP, DST, DSTYK, DTYMK, DVL1, DVL3, DYM, DYNC1H1, DYNC1I2, DYNC2H1, DYNC2LI1, DYRK1A, EARS2, EBF3, EBP, ECEL1, ECHS1, ECM1, EDA, EDAR, EDEM3, EDN1, EDNRA, EDNRB, EED, EEF1A2, EEF1B2, EEF2, EFEMP2, EFHC1, EFNB1, EFTUD2, EGR2, EHMT1, EIF2A, EIF2AK1, EIF2AK2, EIF2AK3, EIF2B4, EIF2B5, EIF2S3, EIF3F, EIF4A2, EIF4A3, EIF4G1, EIF5A, ELAC2, ELFN1, ELK1, ELMO2, ELN, ELOVL4, ELOVL5, ELP2, EMC1, EMC10, EMG1, EML1, EMX2, EN2, ENOX2, ENPP1, ENTPD1, EOGT, EOMES, EP300, EPB41L1, EPCAM, EPG5, EPHA7, EPHB4, EPM2A, EPPK1, EPRS, ERBB3, ERBB4, ERCC1, ERCC2, ERCC3, ERCC4, ERCC5, ERCC6, ERCC6L2, ERCC8, ERF, ERGIC3, ERI1, ERLIN2, ERMARD, ESAM, ESCO2, ESX1, ETFA, ETFB, ETFDH, ETHE1, EVC, EVC2, EXOC2, EXOC7, EXOSC2, EXOSC3, EXOSC8, EXOSC9, EXPH5, EXT1, EXT2, EXTL3, EYA1, EZH2, F5, FA2H, FAAH2, FAH, FAM111A, FAM111B, FAM120C, FAM126A, FAM149B1, FAM160B1, FAM161A, FAM20A, FAM20C, FAM47B, FAM50A, FANCA, FANCB, FANCC, FANCD2, FANCE, FANCF, FANCG, FANCI, FANCL, FANCM, FAR1, FARS2, FARSA, FARSB, FASN, FAT4, FBLN1, FBLN5, FBN1, FBN2, FBP1, FBRSL1, FBXL3, FBXL4, FBXO11, FBXO25, FBXO28, FBXO31, FBXO7, FBXO8, FBXW11, FBXW4, FBXW7, FCSK, FDFT1, FDXR, FEM1B, FEM1C, FEZF1, FGD1, FGD4, FGF10, FGF12, FGF13, FGF14, FGF3, FGF9, FGFR1, FGFR2, FGFR3, FH, FHL1, FIBP, FICD, FIG4, FILIP1, FKBP10, FKBP14, FKBP6, FKBPL, FKRP, FKTN, FLAD1, FLG, FLNA, FLNB, FLT4, FLVCR1, FLVCR2, FMN2, FMR1, FN1, FOLR1, FOSL2, FOXC1, FOXC2, FOXE1, FOXE3, FOXF1, FOXG1, FOXI3, FOXJ1, FOXL2, FOXN1, FOXP1, FOXP2, FOXP3, FOXP4, FOXR1, FOXRED1, FRA10AC1, FRAS1, FREM1, FREM2, FRMD5, FRMD7, FRMPD4, FRRS1L, FRY, FTCD, FTL, FTO, FTSJ1, FUCA1, FUT8, FXN, FXR1, FYCO1, FZD3, FZD5, FZD6, FZR1, G6PC3, GAA, GAB3, GABBR1, GABBR2, GABRA1, GABRA2, GABRA5, GABRB2, GABRB3, GABRD, GABRG1, GABRG2, GABRG3, GABRQ, GAD1, GALC, GALE, GALK1, GALNS, GALNT2, GALT, GAMT, GAN, GAS2L2, GAS8, GATA2, GATA3, GATA4, GATA6, GATAD2B, GATM, GBA, GBA2, GBE1, GCDH, GCH1, GCK, GCSH, GDAP1, GDF1, GDF11, GDF3, GDF5, GDF6, GDI1, GEMIN4, GEMIN5, GFAP, GFER, GFM1, GHR, GIGYF1, GIGYF2, GJA1, GJA3, GJA8, GJB1, GJB2, GJB3, GJC2, GK, GLB1, GLDC, GLDN, GLE1, GLI2, GLI3, GLIS2, GLIS3, GLMN, GLRA1, GLRA2, GLRB, GLS, GLUD1, GLUL, GLYCTK, GM2A, GMNN, GMPPA, GMPPB, GNA11, GNA14, GNAI1, GNAI2, GNAI3, GNAL, GNAO1, GNAQ, GNAS, GNB1, GNB2, GNB3, GNB5, GNE, GNPAT, GNPTAB, GNPTG, GNS, GOLGA2, GON4L, GORAB, GOSR2, GOT2, GPAA1, GPC3, GPC4, GPC6, GPHN, GPR179, GPRASP1, GPSM2, GPT2, GPX4, GRB14, GREB1L, GRHL2, GRHL3, GRIA1, GRIA2, GRIA3, GRIA4, GRID2, GRIK2, GRIN1, GRIN2A, GRIN2B, GRIN2D, GRIP1, GRM1, GRM6, GRM7, GRN, GSPT2, GSS, GSX2, GTF2E2, GTF2H5, GTF2IRD1, GTF3C3, GTPBP2, GTPBP3, GTPBP8, GUCY2C, GUSB, GYS2, GZF1, H3F3A, H3F3B, HAAO, HACD1, HACE1, HADH, HADHA, HADHB, HARS, HARS1, HARS2, HAUS7, HAX1, HCCS, HCFC1, HCN1, HDAC4, HDAC6, HDAC8, HEATR3, HEATR5B, HECTD4, HECW2, HEPACAM, HERC1, HERC2, HESX1, HEXA, HEXB, HGSNAT, HIBCH, HID1, HINT1, HIRA, HIST1H1E, HIST1H2AC, HIST1H4B, HIST1H4C, HIST1H4D, HIST1H4E, HIST1H4F, HIST1H4I, HIST1H4J, HIST3H3, HIVEP2, HK1, HLCS, HMGB1, HMGB3, HMGCL, HMGCS2, HMX1, HNF1B, HNF4A, HNMT, HNRNPA2B1, HNRNPD, HNRNPH1, HNRNPH2, HNRNPK, HNRNPR, HNRNPU, HOXA1, HOXA11, HOXA13, HOXB1, HOXC13, HOXD10, HOXD13, HPD, HPDL, HPGD, HPRT1, HPS1, HPSE2, HR, HRAS, HS2ST1, HS6ST2, HSD17B10, HSD17B4, HSD3B7, HSF4, HSPD1, HSPG2, HTRA2, HTT, HUWE1, HYAL1, HYAL2, HYDIN, HYLS1, IARS, IARS2, IBA57, IDH2, IDS, IDUA, IER3IP1, IFIH1, IFITM5, IFNAR2, IFT122, IFT140, IFT172, IFT27, IFT43, IFT74, IFT80, IGBP1, IGF1, IGF1R, IGF2, IGFBP7, IGHMBP2, IGSF1, IHH, IKBKG, IL11, IL11RA, IL1RAPL1, IL1RAPL2, IL3RA, ILF2, IMPAD1, IMPDH2, INF2, INPP4A, INPP5E, INPP5K, INPPL1, INSR, INTS1, INTS11, INTS6, INTS6L, INTS8, IPO8, IQSEC1, IQSEC2, IQSEC3, IRAK1, IREB2, IRF2BPL, IRF6, IRX5, ISCA2, ITCH, ITFG2, ITGA3, ITGA4, ITGA6, ITGA7, ITGA8, ITGB6, ITIH6, ITPA, ITPR1, IVD, JAG1, JAG2, JAGN1, JAK3, JAKMIP1, JAM3, JARID2, JMJD1C, JPH3, KANK1, KANSL1, KARS, KAT5, KAT6A, KAT6B, KAT8, KATNAL2, KATNB1, KBTBD13, KCNA1, KCNA2, KCNA4, KCNB1, KCNC1, KCNC3, KCND1, KCND2, KCND3, KCNE1, KCNH1, KCNH5, KCNJ10, KCNJ11, KCNJ6, KCNJ8, KCNK12, KCNK3, KCNK4, KCNK9, KCNMA1, KCNN2, KCNN3, KCNQ1, KCNQ2, KCNQ3, KCNQ5, KCNT1, KCNT2, KCTD1, KCTD3, KCTD7, KDELR2, KDM1A, KDM2B, KDM3B, KDM4B, KDM5A, KDM5B, KDM5C, KDM6A, KDM6B, KIAA0586, KIAA1109, KIDINS220, KIF11, KIF14, KIF1A, KIF1B, KIF1BP, KIF1C, KIF21A, KIF21B, KIF22, KIF26B, KIF2A, KIF3B, KIF4A, KIF5A, KIF5B, KIF5C, KIF7, KIRREL3, KIT, KITLG, KLF1, KLF7, KLF8, KLHL15, KLHL20, KLHL21, KLHL34, KLHL4, KLHL40, KLHL7, KMT2A, KMT2B, KMT2C, KMT2D, KMT2E, KMT5B, KNL1, KPNA7, KPTN, KRAS, KRIT1, KRT74, L1CAM, L2HGDH, LAGE3, LAMA1, LAMA2, LAMB1, LAMB2, LAMC3, LAMP2, LARGE1, LARP7, LARS, LARS2, LAS1L, LBR, LDB3, LEF1, LEFTY2, LEMD2, LEMD3, LETM1, LFNG, LGI1, LGI4, LHFPL3, LHX2, LHX3, LHX4, LIAS, LIFR, LIG4, LIMK1, LINGO1, LINGO4, LINS1, LIPN, LIPT1, LIPT2, LITAF, LMAN2L, LMBRD1, LMBRD2, LMNA, LMNB1, LMNB2, LMOD2, LMOD3, LMX1B, LNPK, LONP1, LOXHD1, LRAT, LRBA, LRIG2, LRIT3, LRP1, LRP2, LRP4, LRP5, LRP6, LRPAP1, LRPPRC, LRRC32, LRRC56, LRRC6, LRRK1, LRRK2, LSS, LTBP1, LTBP2, LTBP3, LYRM7, LYST, LZTFL1, LZTR1, MAB21L1, MAB21L2, MACC1, MACF1, MADD, MAF, MAFB, MAGEA11, MAGEB1, MAGEB10, MAGEB2, MAGEC1, MAGEC3, MAGED1, MAGEE2, MAGEL2, MAGI2, MAGIX, MAGT1, MAL, MAMLD1, MAN1B1, MAN2A2, MAN2B1, MAN2C1, MANBA, MAOA, MAOB, MAP1B, MAP2K1, MAP2K2, MAP3K1, MAP3K15, MAP3K7, MAP7D3, MAPK1, MAPK10, MAPK8IP3, MAPKAPK5, MAPRE2, MAPT, MARS2, MASP1, MAST1, MAST4, MAT1A, MATN3, MAU2, MBD5, MBNL3, MBOAT7, MBTPS2, MC2R, MCCC1, MCCC2, MCEE, MCM3AP, MCM9, MCOLN1, MCPH1, MDH2, MECOM, MECP2, MECR, MED11, MED12, MED12L, MED13, MED13L, MED17, MED23, MED25, MED27, MEF2C, MEGF10, MEGF8, MEIS2, MEOX1, MESD, MESP2, MET, METAP1, METTL23, METTL5, MFF, MFN2, MFRP, MFSD2A, MFSD8, MGAT2, MGAT5B, MGP, MIB1, MICU1, MID1, MINPP1, MIR17HG, MIR184, MITF, MKKS, MKS1, MLC1, MLH1, MLYCD, MMAA, MMAB, MMACHC, MMADHC, MMGT1, MMP13, MMP14, MMP15, MMP21, MMUT, MN1, MNX1, MOCS1, MOCS2, MOGS, MORC2, MORC4, MPC2, MPDU1, MPDZ, MPI, MPLKIP, MPP5, MPV17, MPZ, MRAP, MRAS, MRE11, MRPS2, MRPS22, MRPS34, MRTFB, MSI1, MSL2, MSL3, MSMO1, MSX1, MSX2, MT-ATP6, MTF1, MTFMT, MTHFR, MTHFS, MTM1, MTMR1, MTMR14, MTMR2, MTMR8, MT-ND1, MT-ND4, MTO1, MTOR, MTPAP, MTR, MTRFR, MTRR, MTSS2, MT-TK, MT-TL1, MTTP, MT-TP, MVK, MXRA5, MYBPC1, MYCBP2, MYCN, MYF5, MYH10, MYH11, MYH3, MYH6, MYH8, MYH9, MYL1, MYLK, MYLPF, MYO18B, MYO1D, MYO1G, MYO1H, MYO5A, MYO5B, MYO7A, MYOC, MYOCD, MYPN, MYRF, MYSM1, MYT1, MYT1L, NAA10, NAA15, NAA20, NACC1, NADK2, NADSYN1, NAE1, NAGA, NAGLU, NAGS, NALCN, NANS, NAPB, NARS, NARS2, NAXD, NAXE, NBAS, NBEA, NBN, NCAPD2, NCAPD3, NCAPG2, NCAPH, NCDN, NCKAP1, NCOR1, NDE1, NDN, NDNF, NDP, NDRG1, NDST1, NDUFA1, NDUFA10, NDUFA11, NDUFA12, NDUFA2, NDUFA6, NDUFA8, NDUFA9, NDUFAF1, NDUFAF2, NDUFAF3, NDUFAF5, NDUFAF8, NDUFB11, NDUFB3, NDUFB7, NDUFB8, NDUFS1, NDUFS2, NDUFS3, NDUFS4, NDUFS7, NDUFS8, NDUFV1, NDUFV2, NEB, NECAB2, NECAP1, NECTIN1, NECTIN4, NEDD4L, NEFL, NEK1, NEK8, NEMF, NEU1, NEUROD2, NEUROG1, NEXMIF, NF1, NFASC, NFE2L2, NFIA, NFIB, NFIX, NFU1, NGF, NGLY1, NHEJ1, NHLRC1, NHLRC2, NHP2, NHS, NIPA1, NIPBL, NKAP, NKX2-1, NKX2-5, NKX3-2, NKX6-2, NLGN3, NLGN4X, NLRP3, NMNAT1, NODAL, NOG, NONO, NOP10, NOP56, NOTCH1, NOTCH2, NOTCH3, NOVA2, NPC1, NPC2, NPHP1, NPHP3, NPHP4, NPHS1, NPHS2, NPM1, NPR2, NPR3, NR1I3, NR2F1, NR2F2, NR4A2, NR5A1, NRAS, NRCAM, NRK, NRROS, NRXN1, NRXN2, NRXN3, NSD1, NSD2, NSDHL, NSF, NSMCE3, NSRP1, NSUN2, NT5C2, NT5C3A, NTM, NTNG1, NTNG2, NTRK1, NTRK2, NUBPL, NUDT2, NUP107, NUP133, NUP188, NUP214, NUP54, NUP62, NUP85, NUS1, NXF4, NXF5, NYX, OBSL1, OCLN, OCRL, ODAD1, ODAD2, ODAD3, ODAD4, ODAPH, ODC1, ODF2L, OFD1, OGDH, OGDHL, OGT, ONECUT1, OPA3, OPHN1, OR5M1, ORC1, ORC4, ORC6, OSGEP, OTC, OTOGL, OTUD5, OTUD6B, OTUD7A, OTULIN, OTX2, OXCT1, OXR1, P2RY4, P2RY8, P3H1, P4HB, P4HTM, PABPC1, PABPC5, PACS1, PACS2, PAFAH1B1, PAH, PAK1, PAK3, PALB2, PAM16, PAN2, PANK2, PAPSS2, PARK7, PARN, PARP1, PARP6, PASD1, PAX1, PAX2, PAX3, PAX6, PAX7, PAX8, PAX9, PBRM1, PBX1, PC, PCARE, PCBD1, PCBP2, PCCA, PCCB, PCDH10, PCDH12, PCDH19, PCDHGC4, PCGF2, PCLO, PCNT, PCYT1A, PCYT2, PDCD10, PDCD6IP, PDE10A, PDE4D, PDE6D, PDE6G, PDE6H, PDGFB, PDGFRB, PDHA1, PDHB, PDHX, PDIA6, PDP1, PDSS1, PDSS2, PDYN, PDZD8, PECR, PEPD, PET100, PEX1, PEX10, PEX11B, PEX12, PEX13, PEX14, PEX16, PEX19, PEX2, PEX26, PEX3, PEX5, PEX6, PEX7, PGAP1, PGAP2, PGAP3, PGK1, PGM1, PGM2L1, PGM3, PGRMC1, PHACTR1, PHC1, PHF10, PHF14, PHF21A, PHF5A, PHF6, PHF8, PHGDH, PHIP, PHKA1, PHKA2, PHKG2, PHOX2B, PI4KA, PIBF1, PIDD1, PIEZO1, PIEZO2, PIGA, PIGB, PIGC, PIGF, PIGG, PIGH, PIGK, PIGL, PIGM, PIGN, PIGO, PIGP, PIGQ, PIGS, PIGT, PIGU, PIGV, PIGW, PIGY, PIH1D3, PIK3C2A, PIK3C3, PIK3CA, PIK3R1, PIK3R2, PIN4, PINK1, PIP5K1C, PISD, PITRM1, PITX1, PITX2, PITX3, PJA1, PKD1L1, PKHD1, PLA2G6, PLAA, PLAG1, PLCB1, PLCB4, PLCE1, PLCG2, PLCH1, PLCXD1, PLEC, PLEKHG2, PLK1, PLK4, PLOD1, PLOD2, PLOD3, PLP1, PLPBP, PLXNA1, PLXNA2, PLXNB3, PLXND1, PMM2, PMP22, PMPCA, PMPCB, PMS2, PNKD, PNKP, PNP, PNPLA1, PNPLA2, PNPLA6, PNPO, PNPT1, POC1A, POC1B, POGLUT1, POGZ, POLA1, POLD1, POLE, POLG, POLR1A, POLR1C, POLR1D, POLR2A, POLR3A, POLR3B, POLR3GL, POLRMT, POMGNT1, POMGNT2, POMK, POMP, POMT1, POMT2, PORCN, POT1, POU1F1, POU3F2, POU3F3, POU4F1, PPA2, PPFIBP1, PPIL1, PPM1D, PPOX, PPP1CB, PPP1R12A, PPP1R13L, PPP1R15B, PPP1R1B, PPP1R21, PPP1R3F, PPP2CA, PPP2R1A, PPP2R2B, PPP2R5D, PPP3CA, PPT1, PQBP1, PRDM12, PRDM13, PRDM15, PRDM6, PRDX3, PRDX4, PREPL, PRICKLE1, PRICKLE2, PRICKLE3, PRIM1, PRKACA, PRKACB, PRKAR1A, PRKAR1B, PRKCG, PRKD1, PRKG2, PRKN, PRKRA, PRMT7, PRMT9, PRODH, PROP1, PRORP, PROSER1, PROX2, PRPF8, PRPS1, PRR12, PRRG1, PRRG3, PRRT2, PRRX1, PRSS12, PRSS56, PRUNE1, PRX, PSAP, PSAT1, PSEN1, PSMA7, PSMB8, PSMC1, PSMC3, PSMC5, PSMD10, PSMD12, PSPH, PTCH1, PTCHD1, PTDSS1, PTEN, PTF1A, PTH, PTH1R, PTHLH, PTPA, PTPN11, PTPN14, PTPN21, PTPN23, PTPN4, PTPRF, PTRH2, PTRHD1, PTS, PUDP, PUF60, PUM1, PURA, PUS1, PUS3, PUS7, PXDN, PYCR1, PYCR2, PYGL, PYROXD1, QARS, QDPR, QKI, QRICH1, RAB11A, RAB11B, RAB14, RAB18, RAB23, RAB27A, RAB39B, RAB3GAP1, RAB3GAP2, RAB40AL, RAB5C, RAB5IF, RABGAP1, RABL6, RAC1, RAC3, RAD21, RAD50, RAD51, RAD51C, RAF1, RAI1, RALA, RALGAPA1, RALGDS, RANBP17, RANBP2, RAP1B, RAP1GDS1, RAPGEF1, RAPSN, RARB, RARS, RARS2, RASA1, RAX, RBBP8, RBFOX1, RBL2, RBM10, RBM28, RBM8A, RBPJ, RBSN, RECQL4, REEP1, REEP2, RELN, RENBP, RERE, REST, RET, RETREG1, RFT1, RFX3, RFX4, RFX6, RFX7, RGN, RGS7, RHEB, RHOBTB2, RIC1, RIMS1, RIMS2, RIN2, RING1, RINT1, RIPK4, RIT1, RLIM, RMI1, RMND1, RMRP, RNASEH2A, RNASEH2B, RNASEH2C, RNASET2, RNF113A, RNF125, RNF13, RNF135, RNF168, RNF216, RNF220, RNPC3, RNU12, RNU4ATAC, RNU7-1, ROBO1, ROBO3, ROBO4, ROGDI, ROR2, RORA, RORB, RPE65, RPGR, RPGRIP1, RPGRIP1L, RPIA, RPL10, RPL11, RPL13, RPL26, RPS19, RPS23, RPS26, RPS6KA3, RRAS, RRAS2, RRM1, RRM2B, RSPH1, RSPH3, RSPO2, RSPO4, RSPRY1, RSRC1, RTEL1, RTL9, RTN2, RTN4IP1, RTTN, RUBCN, RUNX2, RUSC2, RXYLT1, RYR1, RYR2, RYR3, SACS, SALL1, SALL4, SAMD9, SAMD9L, SAMHD1, SARS, SARS2, SATB1, SATB2, SBDS, SBF1, SBF2, SC5D, SCAF4, SCAMP5, SCAPER, SCARB2, SCARF2, SCN11A, SCN1A, SCN1B, SCN2A, SCN3A, SCN4A, SCN8A, SCN9A, SCNM1, SCO1, SCO2, SCRIB, SCUBE3, SCYL1, SDCCAG8, SDHA, SDHAF1, SEC23A, SEC23B, SEC24D, SEC31A, SEC61A1, SECISBP2, SELENOI, SELENON, SEMA3A, SEMA3E, SEMA6B, SEPSECS, SERAC1, SET, SETBP1, SETD1A, SETD1B, SETD2, SETD5, SETDB2, SETX, SF3B4, SFXN4, SGCA, SGCE, SGPL1, SGSH, SGSM3, SH3BP2, SH3PXD2B, SH3TC2, SHANK1, SHANK2, SHANK3, SHH, SHMT2, SHOC2, SHOX, SHQ1, SHROOM2, SHROOM3, SHROOM4, SIAH1, SIGMAR1, SIK1, SIL1, SIM1, SIN3A, SIN3B, SIX1, SIX3, SIX5, SIX6, SKI, SKIV2L, SLC10A7, SLC12A2, SLC12A5, SLC12A6, SLC13A1, SLC13A5, SLC16A2, SLC17A5, SLC18A2, SLC19A3, SLC1A1, SLC1A2, SLC1A4, SLC20A2, SLC22A5, SLC24A1, SLC24A4, SLC25A1, SLC25A12, SLC25A13, SLC25A15, SLC25A19, SLC25A20, SLC25A22, SLC25A24, SLC25A26, SLC25A38, SLC25A4, SLC25A42, SLC25A53, SLC25A6, SLC26A2, SLC26A9, SLC27A4, SLC2A1, SLC2A10, SLC2A2, SLC30A7, SLC30A9, SLC31A1, SLC32A1, SLC33A1, SLC35A1, SLC35A2, SLC35A3, SLC35B2, SLC35C1, SLC35D1, SLC35F1, SLC37A4, SLC38A3, SLC39A13, SLC39A14, SLC39A8, SLC45A1, SLC46A1, SLC4A1, SLC4A11, SLC4A4, SLC52A2, SLC52A3, SLC5A2, SLC5A5, SLC5A6, SLC5A7, SLC6A1, SLC6A17, SLC6A19, SLC6A3, SLC6A4, SLC6A5, SLC6A8, SLC6A9, SLC7A7, SLC9A6, SLC9A7, SLC9A9, SLF2, SLIRP, SLX4, SMAD2, SMAD3, SMAD4, SMAD6, SMARCA1, SMARCA2, SMARCA4, SMARCA5, SMARCAL1, SMARCB1, SMARCC1, SMARCC2, SMARCD1, SMARCD2, SMARCD3, SMARCE1, SMC1A, SMC3, SMC5, SMCHD1, SMG8, SMG9, SMO, SMOC1, SMOC2, SMPD1, SMPD4, SMS, SNAP25, SNAP29, SNAPC4, SNCA, SNIP1, SNORD118, SNRPB, SNRPE, SNTG1, SNX14, SNX27, SNX3, SOBP, SON, SOS1, SOS2, SOX10, SOX11, SOX17, SOX2, SOX3, SOX4, SOX5, SOX6, SOX9, SPAG1, SPARC, SPART, SPAST, SPATA5, SPATA5L1, SPECC1L, SPEG, SPEN, SPG11, SPG21, SPG7, SPOP, SPR, SPRED1, SPRED2, SPRTN, SPRY1, SPRY3, SPTAN1, SPTBN1, SPTBN2, SPTBN4, SPTLC1, SPTLC2, SRCAP, SRD5A3, SREBF2, SRGAP3, SRP54, SRPX2, SRRM2, SRSF1, SRY, SSR4, ST14, ST3GAL3, ST3GAL5, STAB2, STAC3, STAG1, STAG2, STAMBP, STAR, STARD8, STAT1, STAT2, STAT5B, STIL, STIM1, STN1, STRA6, STRADA, STS, STT3A, STT3B, STUB1, STX11, STX1A, STX1B, STX3, STXBP1, SUCLA2, SUCLG1, SUFU, SUMF1, SUMO1, SUOX, SUPT16H, SURF1, SUZ12, SVBP, SYN1, SYNCRIP, SYNE1, SYNE2, SYNGAP1, SYNJ1, SYP, SYT1, SYT14, SYT2, SYTL4, SYTL5, SZT2, TAB2, TAC3, TACO1, TACR3, TAF1, TAF13, TAF1C, TAF2, TAF4, TAF6, TAF7L, TAF8, TAFAZZIN, TANC2, TANGO2, TAOK1, TAPT1, TARDBP, TARS, TASP1, TAT, TAZ, TBC1D20, TBC1D23, TBC1D24, TBC1D2B, TBC1D7, TBC1D8B, TBCD, TBCE, TBCK, TBL1XR1, TBP, TBR1, TBX1, TBX15, TBX18, TBX20, TBX22, TBX3, TBX4, TBX5, TBXAS1, TCEAL1, TCEAL3, TCF12, TCF20, TCF4, TCF7L2, TCN2, TCOF1, TCP10L2, TCTN1, TCTN2, TCTN3, TDP2, TDRD7, TECPR2, TECR, TEFM, TEK, TELO2, TENM1, TENM3, TEPSIN, TERC, TERT, TET3, TFAP2A, TFAP2B, TFB2M, TFE3, TFG, TFRC, TGDS, TGFB1, TGFB2, TGFB3, TGFBR1, TGFBR2, TGIF1, TGM6, TH, THAP1, THG1L, THOC2, THOC6, THRA, THRB, THUMPD1, TIAM1, TIMM50, TIMM8A, TINF2, TK2, TKFC, TKT, TKTL1, TLK2, TLL1, TLR8, TM4SF20, TMCO1, TMEM106B, TMEM114, TMEM126B, TMEM132E, TMEM135, TMEM147, TMEM163, TMEM165, TMEM199, TMEM216, TMEM218, TMEM222, TMEM231, TMEM237, TMEM240, TMEM251, TMEM260, TMEM63A, TMEM63B, TMEM63C, TMEM67, TMEM70, TMEM94, TMLHE, TMPRSS6, TMPRSS9, TMTC3, TMX2, TNFRSF13B, TNIK, TNKS2, TNNT3, TNPO2, TNR, TNRC6B, TOE1, TOGARAM1, TOMM70, TOP3A, TOR1A, TP53RK, TP63, TP73, TPH2, TPK1, TPM2, TPM3, TPP1, TPP2, TPR, TPRKB, TRA2B, TRAF7, TRAIP, TRAK1, TRAPPC10, TRAPPC11, TRAPPC12, TRAPPC2, TRAPPC2L, TRAPPC4, TRAPPC6A, TRAPPC6B, TRAPPC9, TREX1, TREX2, TRHR, TRIM32, TRIM37, TRIM8, TRIO, TRIP11, TRIP12, TRIP13, TRIP4, TRIT1, TRMT1, TRMT10A, TRMT10C, TRNT1, TRPC5, TRPM1, TRPM3, TRPS1, TRPV3, TRPV4, TRPV6, TRRAP, TSC1, TSC2, TSC22D3, TSEN15, TSEN2, TSEN34, TSEN54, TSFM, TSHB, TSHR, TSHZ1, TSPAN7, TSPAN8, TSPEAR, TSPOAP1, TTBK2, TTC12, TTC19, TTC37, TTC5, TTC7A, TTC8, TTI1, TTI2, TTN, TTPA, TTR, TUBA1A, TUBA8, TUBAL3, TUBB, TUBB2A, TUBB2B, TUBB3, TUBB4A, TUBG1, TUBGCP2, TUBGCP4, TUBGCP6, TUFM, TUSC3, TWIST1, TWIST2, TXNL4A, TYR, TYRP1, U2AF2, UBA5, UBAP2L, UBE2A, UBE2T, UBE2U, UBE3A, UBE3B, UBE3C, UBE4A, UBR1, UBR4, UBR7, UBTF, UFC1, UFM1, UFSP2, UGDH, UGP2, UGT1A1, UHRF1, UMPS, UNC13A, UNC45A, UNC45B, UNC80, UPB1, UPF1, UPF3B, UQCRB, UQCRFS1, UQCRQ, UROC1, UROS, USB1, USP14, USP18, USP27X, USP7, USP9X, UTP14A, UTP4, UVSSA, VAC14, VAMP1, VAMP2, VAMP7, VANGL1, VARS, VARS2, VCP, VDR, VIP, VIPAS39, VLDLR, VPS11, VPS13B, VPS26C, VPS33B, VPS35, VPS35L, VPS41, VPS4A, VPS50, VPS51, VPS53, VRK1, VSX2, WAC, WARS, WARS2, WASF1, WASHC4, WASHC5, WDFY3, WDPCP, WDR11, WDR13, WDR19, WDR26, WDR34, WDR35, WDR37, WDR4, WDR45, WDR45B, WDR5, WDR60, WDR62, WDR73, WDR81, WDR83OS, WFS1, WIPI2, WNK3, WNT1, WNT10B, WNT3, WNT4, WNT5A, WNT7A, WNT7B, WRAP53, WRN, WT1, WWC3, WWOX, XIAP, XIST, XK, XKRX, XPA, XPC, XPNPEP3, XRCC4, XYLT1, XYLT2, YAP1, YARS, YARS2, YIF1B, YIPF5, YRDC, YWHAE, YWHAG, YWHAZ, YY1, ZBTB11, ZBTB16, ZBTB18, ZBTB20, ZBTB24, ZBTB40, ZBTB47, ZBTB7A, ZC3H14, ZC4H2, ZCCHC12, ZCCHC8, ZDHHC15, ZDHHC9, ZEB1, ZEB2, ZFHX3, ZFHX4, ZFP57, ZFPM2, ZFX, ZFYVE19, ZFYVE26, ZIC1, ZIC2, ZIC3, ZMIZ1, ZMPSTE24, ZMYM2, ZMYM3, ZMYM6, ZMYND10, ZMYND11, ZMYND12, ZMYND8, ZNF142, ZNF148, ZNF292, ZNF335, ZNF407, ZNF41, ZNF425, ZNF462, ZNF526, ZNF592, ZNF599, ZNF668, ZNF674, ZNF699, ZNF711, ZNF713, ZNF750, ZNF81, ZSWIM6, RBBP5, PLXNB2*
